# The Daughter Cyst

**DOI:** 10.5811/cpcem.2016.11.32453

**Published:** 2017-01-23

**Authors:** Adrianna Kyle, Maxwell Cooper, Katrina Destree, Lauren Oliveira, Matthew Lawrence

**Affiliations:** Naval Medical Center Portsmouth, Department of Emergency Medicine, Portsmouth, Virginia

## CASE REPORT

A 35-year-old woman, gravida 4 para 3 estimated at 9+1 weeks gestational age by uncertain last menstrual period, presented with vaginal bleeding. She endorsed unprotected intercourse eight weeks prior and took Plan B One-Step (“morning-after pill”) within 48 hours. Quantitative human chorionic gonadotropin (hcg) resulted at 30 mIU/ml. Bedside transabdominal ultrasound was concerning for ectopic pregnancy (Video). Is this ectopic pregnancy?

Radiology performed a transvaginal ultrasound, which was negative for intrauterine or ectopic pregnancy. Obstetric consultants were uncertain if the structure seen at bedside represented an ectopic (Image). Radiology believed that this structure, which appeared initially to be an extra-uterine yolk sac, was instead a daughter cyst. The patient was well appearing and hemodynamically stable. She was discharged with ectopic precautions and a repeat 48-hour quantitative hcg, which ultimately trended to 0.

## DISCUSSION

The daughter cyst sign indicates an uncomplicated ovarian cyst.^1^ It is a peripherally based simple cyst within a larger simple cyst.^2^ On pathology, it represents a stimulated ovarian follicle.^2^ This sonographic finding must be differentiated from an ectopic pregnancy in any woman with the potential to become pregnant.^1^ Previously documented cases in the literature

are limited to case reports involving pediatric females with McCune –Albright Syndrome and precocious puberty, as well as fetuses with incidental cysts mimicking ectopic pregnancy.^1^,^2^ Differentiation can be made with a quantitative hcg, which is negative in the case of the daughter cyst. Differentiation is also made sonographically with a “ring of fire” sign on the structure’s periphery, indicative of increased color Doppler flow to an ectopic pregnancy, whereas a true daughter cyst has no increased flow.^1^ In the case of our patient, she had a down-trending hcg due to recent administration of Plan B for unwanted pregnancy, and an incidentally noted daughter cyst that was initially concerning for an ovarian pregnancy.

## Supplementary material

Video Point-of-care transabdominal ultrasound video clip demonstrating the uterus and daughter cyst in the axial plane.

## Figures and Tables

**Image:**
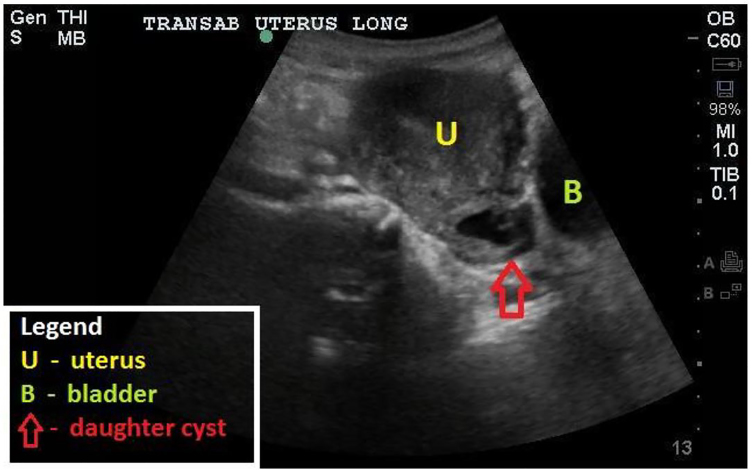
Point-of-care transabdominal ultrasound demonstrating the uterus and daughter cyst in the sagittal plane.
